# Supplementary figures and images for: Artificial intelligence system can achieve comparable results to experts for bone age assessment of Chinese children with abnormal growth and development
Source: PeerJ. 2020 Apr 1;8:e8854. doi: 10.7717/peerj.8854 (PMC7127473; doi:10.7717/peerj.8854)

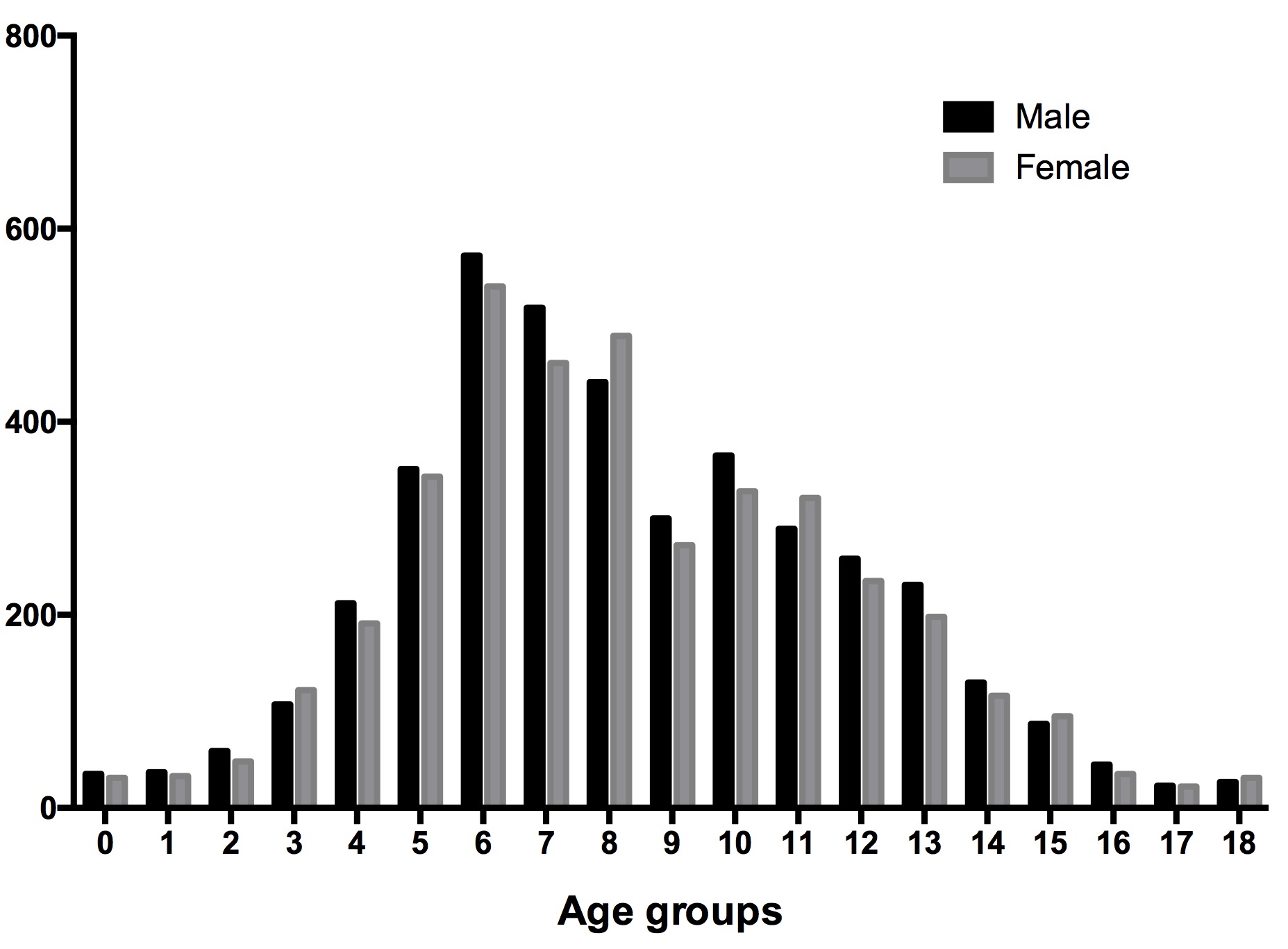

Supplement: Supplemental Information 3 [file peerj-08-8854-s003.jpg]
