# Supplementary material for: Artificial intelligence system can achieve comparable results to experts for bone age assessment of Chinese children with abnormal growth and development
Source: PeerJ. 2020 Apr 1;8:e8854. doi: 10.7717/peerj.8854 (PMC7127473; doi:10.7717/peerj.8854)
Supplement: Supplemental Information 4 [file peerj-08-8854-s004.docx]

**Table S1 The sex characteristics of data set used to develop the algorithm**

|  | **Male** | **Female** | **Total** |
| --- | --- | --- | --- |
|  |  |  |  |
| **Training set** | 3545 | 3455 | 7000 |
| **Validation set** | 417 | 343 | 760 |
| **Test set** | 125 | 115 | 240 |
